# Supplementary material for: Digital literacy: The catalyst for credit access and income growth in rural households
Source: PLoS One. 2026 Feb 13;21(2):e0341326. doi: 10.1371/journal.pone.0341326 (PMC12904465; doi:10.1371/journal.pone.0341326)
Supplement: Attachment — Submitted filename: Response to Reviewers.docx [file pone.0341326.s003.docx]

Response to Reviewers

Dear Reviewers,

We would like to extend our sincere gratitude to all of you for your respective valuable comments and constructive suggestions on our manuscript. These insights have been instrumental in enhancing the quality and rigor of our work, and we have carefully addressed each comment from every reviewer in the revised version, as detailed below.

**1.For one of the reviewers’ comments, we have prepared a summary response. This is because the comments of this reviewer primarily focus on questioning the validity of Ordinary Least Squares (OLS) and emphasizing the importance of instrumental variable regression.**

**We first present the original comments of this reviewer, followed by our summary response structured in the logical order of instrumental variable regression:**

Original Comments of the Reviewer：(1)I don't think OLS is the right model to study this research question. While the authors correctly refer to it as a "benchmark model," the main focus of the paper should be on the IV regression. I won't even introduce the equation for OLS in Section 3.

(2) I don't believe that the aggregate IV is the right model either. This variable, in this paper's empirical setting, unnecessarily creates a network problem because a person's digital literacy (and socioeconomic status) will be undoubtedly affected by the neighbors' digital literacy and socioeconomic status, unless the authors are ready to claim that individuals in this paper's empirical setting are all very isolated (but how so??). Charles Manski has a famous "reflection problem" paper entailing this, so I don't think that this IV is an appropriate one here.

The least the authors can do is to design a historically driven aggregate IV, which is still problematic but is at least more plausible than using a current measure to quantify a current level of literacy (and other outcomes). For example, some historical measures of infrastructure, human capital, or average household characteristics dated back to decades ago from the statistics yearbook. This way, the authors make a more plausible IV -- but keep in mind that even if the authors do so, it would be extremely important to contextually convince readers why such an IV is plausible, and it requires careful explanation for the Chinese context. I know very little about China, so I'd rather stop commenting here.

I believe that the editor himself has a few papers about it: see his "Acculturational homophily" (Econ of Ed Review) and Identifying ethnic occupational segregation (J of Pop Econ). I hope he can further navigate the authors on this matter.

(3) Relatedly, the authors did not mention anything about the OLS bias. Of course the OLS is biased. But how biased? Why is it upward (or downward) biased? What factors drive this bias, and is there an indirect way to evaluate it? The authors did a poor job analyzing the exact way endogeneity emerges in the dataset.

(4) I would just drop PSM. It doesn't help much solve endogeneity problems.

**We now present our response to this reviewer’s comments:**

First, this revision has clarified the issue of result bias prone to be caused by Ordinary Least Squares (OLS).

Second, the reviewer argues that the aggregate instrumental variable (IV) used in the original manuscript is not an appropriate instrumental variable. Although this IV has been widely adopted by Chinese scholars when studying the digital literacy of rural residents in China, we have adopted the reviewer’s suggestion in this revision: we have removed this instrumental variable and will continue to search for a more suitable one.

Third, the focus of this revision lies in the instrumental variable regression method. The section on instrumental variable revision draws on the methodological approach from Dafeng Xu’s literature and adopts historical exogenous variables as instrumental variables.

Below is an explanation of the specific content of the revisions :

The baseline OLS estimation may be subject to two types of endogeneity biases that could lead to biased results. First, high-income farmers are more likely to purchase smart devices and participate in paid digital training, which may result in reverse causality bias. Second, there may be omitted variable bias.

This study employs the Instrumental Variable (IV) approach to address reverse causality bias. The IV strategy in this study draws on the design of historical exogenous variables proposed by Dafeng Xu (2017), constructing IVs based on the dual exogenous structure of geographical endowments and historical policies. The first instrumental variable (IV1) selected in this study is the distance from farmers' residences to the administrative center. The location of township-level administrative centers is determined by county-level master plans and has remained stable for a long time since the "township merger policy" in the 1990s. The farther the distance to the township administrative center, the lower the convenience for farmers to access offline services, and the higher their dependence on digital infrastructure and online digital resources—this indirectly affects digital literacy. The second instrumental variable (IV2) is whether a village has 5G coverage. In 2019, national-level planning launched 5G planning and construction, which did not take farmers' income as the criterion for 5G coverage, making it an exogenous variable derived from historical policies. Villages with 5G coverage are more conducive to farmers participating in online digital activities to improve their digital literacy.

The results of the Two-Stage Least Squares (2SLS) estimation are presented in Table 4.The first-stage regression results show: (1) The overall F-statistic = 14.95 (P<0.001), which is greater than the empirical critical value of 10, indicating that the two IVs jointly have a significant explanatory power for digital literacy, satisfying the strong correlation assumption; (2) The Cragg-Donald Wald F-statistic is 23.67. For the scenario of "1 endogenous variable and 2 instrumental variables," the Stock-Yogo 10% maximum IV size critical value is 19.93. The current F-statistic exceeds this critical value, avoiding estimation bias caused by weak instruments; (3) The Sargan statistic = 1.23 (P=0.267>0.05), failing to reject the null hypothesis that "all instrumental variables are exogenous." Thus, the exogeneity test of the IVs is passed.

The 2SLS estimation results show that the IVs effectively correct endogeneity bias, and the core conclusions are robust. The coefficient of digital literacy is 1.520, which is significant at the 1% level. Compared with the baseline OLS, the coefficient of digital literacy in 2SLS is significantly larger—this verifies the conjecture that "reverse causality leads to OLS underestimating the true effect," and the IVs correct this bias.

The second potential issue is omitted variable bias. The author used the Propensity Score Matching (PSM) method to address this bias in the initial draft. However, considering that the reviewers suggested removing this method, the author has omitted the process of the PSM method in this revision and only reports the results of the PSM method.

**The other reviewer suggested regarding the original manuscript:**

2.The equal - weight assignment assumes all indicators contribute equally to digital literacy. But this assumption lacks a solid theoretical basis and isn't verified by actual data. It may underestimate the weight of some key areas and make the measurement results fail to reflect farmers' real digital literacy. Maybe you can consider combined weighting methods like game - theory or grey relational analysis - TOPSIS.

In response to the revision requirements, we adopted two methods to address the aforementioned issue, with details as follows:

**For Method 1**, we used the entropy weight method to construct the Digital Literacy (DL) indicator. The core objective was to determine indicator weights based on the "information content" or "variation degree" of each indicator’s data—specifically, indicators with greater variation carry more information and thus receive higher weights. The implementation process involved four sequential steps: standardizing the original data, calculating the information entropy of each indicator, deriving the differentiation coefficient from the information entropy, and obtaining normalized weights. This is a data-driven objective weighting method, which essentially answers the question: "Which indicator has higher discriminative power in the existing dataset?"

**For Method 2**, we constructed a new DL indicator using the TOPSIS method based on entropy weights, aiming to evaluate the relative closeness of each sample to the "ideal solution." The implementation included four key steps: First, weight calculation was conducted using the entropy weight method, which was identical to Method 1 and yielded a set of objective weights based on data variability. Second, we identified the optimal value (positive ideal solution) and worst value (negative ideal solution) of each indicator across all samples. Third, we computed the distance from each sample to the positive ideal solution ((D^+^)) and to the negative ideal solution ((D^-^)). Fourth, the relative closeness coefficient (i.e., the DL indicator) was calculated using the formula: (DL = D^-^/ (D^+^ + D^-^)). Notably, the DL indicator derived from this method measures a sample’s "comprehensive coordination" or "similarity to the optimal pattern" and is highly sensitive to indicator shortcomings—poor performance in any single indicator will significantly increase the sample’s distance from the positive ideal solution, leading to a sharp drop in its DL score.

Regarding the regression results: When the DL indicator from Method 1 was incorporated into the regression model, DL exerted a statistically significant impact on farm households’income. In contrast, the DL indicator from Method 2 showed no statistically significant impact on farm households’income. Two potential explanations for this discrepancy are as follows: (1) The TOPSIS method itself is more sensitive to data distribution and outliers; (2) The dependent variable (farm households’income) relies more on the "comprehensive level" of DL than its "balanced structure," which aligns with the theoretical analysis of the relationship between farm households’income and DL (farm households’income is primarily determined by the comprehensive level of their digital literacy).

Given this, Method 1 is more suitable for this study, and we have reported the results of Method 1 in Table 6.

3.In first the paragraph of the introduction, the focus on rural digital development appears abruptly at the end.

In the Introduction section, we have supplemented the research background and research significance of this study, and elaborated on the importance of digital literacy and access to credit for farm households’income, as well as the potential of digital literacy in alleviating credit constraints.

4.Hypothesis 3 refers to differences due to gender levels, but the last paragraph of Section 2.3 mainly argues that the growth of the digital economy has significantly increased overall female employment and income, which is irrelevant to the hypothesis.

In the current revision of the manuscript, we have supplemented specific explanations regarding traditional gender norms and the pattern of gender division of labor in rural China in Section 2.3. Specifically, this supplementary content aims to provide a detailed elaboration on the potential gender heterogeneity in the impact of digital literacy on farmers’ income—by contextualizing the traditional gender-related contexts in rural China, we further clarify why the effect of digital literacy on income may vary between male and female farmers, thereby enhancing the theoretical logic and contextual pertinence of this part of the analysis.

5.The paper positions digital literacy as "a more equitable form of human capital", but how is its fairness reflected? The quantile regression shows digital literacy mostly affects the 90th percentile income farmers. Yet, the paper directly claims "digital literacy promotes fairness", which contradicts the result that "high - income groups benefit more".

We would like to thank the reviewers for pointing out this error, and we have removed the expression "a more equitable form of human capital" from the manuscript.

6.The author conducts surveys on the Party branch secretaries or village committee directors of rural administrative villages. Reliance on reports from these officials may lead to administrative statistical bias or cognitive errors on household - level data. It's suggested that the author add a discussion on the limitations of data collection methods in the paper.

First, we apologize for the inaccuracy in the description of the study's participants. The participants include ordinary villagers, and the manuscript omitted the description of ordinary villagers in the section explaining the study's participants. This revision has supplemented the relevant explanation in Section 3.2.1.

7.The policy recommendations seem vague and lack feasibility.

To address the issues of vagueness and lack of feasibility in the original policy recommendations, the revision focuses on supplementing key elements including implementation entities, specific measures, quantitative standards, and supporting guarantees, with clear clarification on "who will implement", "how to implement", and "how to put into practice".

(1) Digital skill development for farmers: Tiered and categorized training will be jointly conducted by county-level governments in collaboration with local agricultural universities and agricultural-related enterprises, covering the following three aspects: First, training on digital collaboration capabilities. Village-level cooperatives will take charge of online order management; leading enterprises will be responsible for online sales and transportation management; and small-scale farmers will handle offline production. Agricultural universities and enterprises will formulate the full-process collaboration plan and provide corresponding training. Second, training on digital security capabilities. Village-level cooperatives and leading enterprises will deliver case-based teaching to farmers, focusing on identifying agricultural-related telecom fraud and protecting the privacy of agricultural production data. Third, training on digital content integration capabilities. The government will cooperate with leading e-commerce enterprises to develop APPs or websites, which will be used to analyze and release market demand. A unified online platform will integrate all entities in the agricultural product supply chain and sales chain, providing farmers with training and services covering all links of e-commerce.

Meanwhile, a 6-month post-training follow-up mechanism will be established, aiming to increase the usage rate of digital tools among low-income groups by more than 30%.

(2) Connection between digital credit and digital industries: First, expand access to digital credit. The government will promote financial institutions such as rural credit cooperatives and MYbank to launch pro-farmer digital credit, which is a credit loan based on farmers’ production data and the pledge of pre-sold agricultural product orders. Second, guide the targeted investment of credit funds. The government and financial institutions will give priority to supporting the settlement of agricultural product e-commerce industrial parks, the IoT transformation of greenhouse farms, and the rural live-streaming economy. They will also invest funds to support the incubation of farmers’ live-streaming accounts for product promotion.

(3) Improvement of digital literacy among vulnerable groups: First, the government will guide public perception: Through village radio broadcasts, short videos, and door-to-door lectures, it will popularize cases of "increasing income via digital tools", such as stories of local rural women increasing their income by selling handcrafted products through e-commerce. Second, tiered training: For groups with education level of primary school or below, an illustrated Rural Digital Skills Manual (with a function of scanning QR codes to watch practical operation videos) will be developed, and "digital assistance positions" will be set up in village service centers (staffed with college-graduate village officials or volunteers to provide one-on-one guidance). The government and enterprises will launch "Women’s Digital Skills Classes" for rural women, focusing on e-commerce operation of handcrafted products and live-streaming of parent-child-oriented agricultural products.

8.The reference list doesn't directly cite UNESCO's authoritative reports (such as "A Global Framework of Reference on Digital Literacy Skills for Indicator").

In this revision, the specific URL of UNESCO's authoritative report "A Global Framework of Reference on Digital Literacy Skills for Indicator" has been provided in the footnote of Section 3.2.3, allowing readers to directly read and download the report on UNESCO's official website by clicking the URL.

9.Research based on data from a single year is inevitably at risk of being affected by extreme years. The economic policy environment and technological penetration characteristics of 2023 may make the research results time - specific. Although the author has enhanced the reliability of cross - sectional data through rigorous empirical methods, it's necessary to explicitly state the year's limitations in the paper or add time - dimension analysis in follow - up research to strengthen the conclusion's universality.

We would like to thank the reviewers for their comments. In this revision, an explanation of the improvement methods has been added at the end of the paper.Future research plans to adopt appropriate panel data to conduct an in-depth exploration of the temporal impact of digital literacy improvement on residents' income, so as to enhance the universality of the conclusions.

10.Many viewpoints lack literature support. It is recommended to supplement the literature.

In this revision, some references have been added in endnotes, and the URLs of the policy documents have been added in footnotes.

11.Note on Data Privacy

This survey was initiated by the Rural Revitalization Research Institute of Beijing Technology and Business University, and jointly conducted in collaboration with the Department of Agriculture and Rural Affairs of the Ministry of Finance, the Chinese Academy of Fiscal Sciences, and other universities. The Agricultural and Rural Affairs Division of the Provincial Department of Finance was responsible for the distribution and collection of survey materials. As one of the participating parties, we have signed a confidentiality agreement, under which we are not permitted to disclose the survey data from the research report. Any violation of this agreement would subject us to substantial fines and legal consequences.

Once again, we would like to thank all of you for your time, expertise, and thoughtful feedback—your insights have been crucial to refining our manuscript. We hope the revised version addresses all your concerns adequately, and we stand ready to provide further clarification or make additional adjustments should any of you have further queries.
