# Supplementary material for: Digital literacy: The catalyst for credit access and income growth in rural households
Source: PLoS One. 2026 Feb 13;21(2):e0341326. doi: 10.1371/journal.pone.0341326 (PMC12904465; doi:10.1371/journal.pone.0341326)
Supplement: Attachment — Submitted filename: editors notes.docx [file pone.0341326.s002.docx]

Digital Literacy: The Catalyst for Credit Access and Income Growth in Rural Households

**Ying Zhang^1,2,3^, Haozhao Zhen^1^*,Yi Wang^4^**

**1** School of Economics,Beijing Technology and Business University, Beijing 100048, China. **2** School of Economics and Management, Hainan Normal University, Haikou 571158, China. **3** Zhongshang Guoneng Group Co., Ltd., Beijing 101299, China**. 4** School of Labor Economics, Capital University of Economics and Business, Beijing 100070, China.

* Corresponding author

E-mail:1121942124@qq.com(HZ)

# ABSTRACT

This paper constructs a digital literacy assessment framework across seven dimensions and employs data from a 2023 rural survey in China to empirically examine the impact of digital literacy on farm household income. The findings indicate that digital literacy significantly enhances income growth among farm households. Mechanism analysis reveals that digital literacy improves access to credit for farm households, thereby facilitating income growth. The effect of digital literacy on household income varies by group, with the highest marginal contribution to agricultural income among low-income households and to non-agricultural income among middle to high-income households. Digital literacy positively affects income across different genders and educational levels. The findings of this study reveal that digital literacy promotes income growth among different groups of rural households in China through various pathways.

**Keywords:** Digital Literacy; Credit access; Farm household income

# 1. Introduction

The year 2024 marks the 10th anniversary of the strategic objective of building a strong cyber country proposed by the Chinese government departments. The Chinese government departments issued the Key Points for Enhancing the Digital Literacy and Skills of the Entire Population in 2024, which delineates the annual objectives: by the end of 2024, China is poised to ascend to a new level in the development of digital literacy and skills for the entire population, with a more comprehensive system for cultivating digital literacy and skills, comprehensive advancement in the construction of a digital barrier-free environment, and a further narrowing of the digital divide among different groups^[1]^. In 2023, the Chinese government departments issued the Overall Layout Plan for the Construction of Digital China, proposing that by 2025, digital infrastructure will be efficiently interconnected, the scale and quality of data resources will be rapidly enhanced, the value of data elements will be effectively released, and the quality and benefits of the digital economy will be significantly improved. The digital countryside represents a strategic orientation for the revitalization of rural China^[2]^. The Outline for the Development Strategy of Digital Villages issued by Chinese government entities sets strategic objectives: by 2025, significant progress in digital rural construction is to be achieved; by 2035, substantial advancements are expected, including a marked reduction in the urban-rural digital divide and a notable increase in farmers’digital literacy^[3]^. These policy documents indicate that the Chinese government is implementing measures to enhance nationwide digital literacy, fostering digital economy growth and societal digital transformation, with a focus on rural digital advancement.

As China transitions into the digital economy era, new-generation information technologies are increasingly integrated into the modernization of agriculture and rural areas, positioning digital literacy as a pivotal driver of rural household income growth. Households with basic digital proficiency can leverage precision agricultural technologies—such as smartphone-based soil testing applications and drone operation systems—to optimize input allocation. Those with advanced digital capabilities further engage in emerging sectors, including rural tourism marketing, paid agricultural knowledge services, and rural e-commerce management, thereby diversifying income streams.In the digital economy context, the Chinese government has actively advanced digital financial infrastructure development. This initiative gains particular relevance as credit constraints persistently impede the optimization of agricultural production inputs, hinder the extension and value addition of agricultural value chains, and weaken rural households' risk resilience. Digital literacy is emerging as a critical mediator enabling rural households to access digital credit mechanisms and alleviate such constraints[1]. Consequently, digital technology penetration facilitates the integration and optimization of resource allocation in rural regions, fostering economic development and income enhancement. However, the underdeveloped digital literacy and limited adoption of digital applications among Chinese farmers currently impede the full unleashing of digital technology's potential benefits.

The Chinese Academy of Social Sciences’ 2021 report Survey and Analysis of Digital Literacy in Rural China reveals that overall digital literacy among Chinese residents is low, with rural residents scoring 37.5% lower than urban counterparts in digital literacy, exhibiting a 27.7% gap in digital income-generating capacity, and a 43.2% deficit in digital safety awareness. Among various occupations, farmers have the lowest digital literacy scores, 57% below the average^[4]^. Despite the widespread penetration of smartphones in rural areas, the capacity for their use by rural residents is rapidly converging with urban levels. These findings suggest that the Chinese government is implementing measures to boost nationwide digital literacy, driving the digital transformation of the economy and rural development. While digital devices are widely available in rural areas, the value of digital literacy in enhancing rural incomes remains to be fully realized.

Existing literature on the relationship among digital literacy, credit access, and rural household income primarily focuses on three aspects: (1) the impact of digital-related factors on rural household income. Some scholars have pointed out that there are various conclusions, such as positive correlation [2-3], nonlinear correlation [4], and irrelevance between the popularization and application of digital technology and individuals’income[5].China has made significant strides in digital rural development, harnessing internet technologies to introduce new economic models in rural areas, such as platform economies and flow economies. These innovations have spurred economic growth and ultimately contributed to income growth for rural residents[6-7]. Scholars utilizing survey data from rural China in 2020 and 2021 have found that enhanced digital literacy enables rural households to more effectively apply advanced technologies, access market information, expand sales channels, increase employment opportunities, broaden social networks, and optimize asset allocation[8], resulting in significant income growth effects.

(2) Regarding the relationship between household borrowing and income, it is primarily categorized into three effects: positive promotion, negative constraint, and threshold effects. Firstly, in the perspective of positive promotion of income growth through household borrowing, Hartarska et al. (2015) found a positive correlation between commercial bank lending in rural areas and regional economic development in the U.S. rural credit market[9]. Gu (2021) also drew a similar conclusion using 2018 survey data from Chinese households, indicating that borrowing significantly enhances household income[10].Secondly, in the research perspective that household borrowing constrains income growth among rural households, many scholars have highlighted the phenomenon of “elite capture” in China’s rural formal credit market, arguing that rural credit offers limited assistance in improving the income of impoverished households[11]. Luan et al. (2016) similarly found in their study of Vietnam’s rural credit market that households with better economic conditions benefit more from rural credit[12]. Hu et al. (2016) suggest that informal micro-lending can significantly increase rural household income and alleviate rural poverty, whereas formal rural finance may have a negative effect on household income, exacerbating rural poverty[13]. Du et al. (2017) analyzed panel data from 29 provinces in mainland China from 2005 to 2015 and discovered that rural credit exhibits a threshold characteristic in promoting farmers’ income growth, inhibiting income increase when farmers’income is below a certain threshold[14]. Gu et al. (2019) proposed that once the threshold value (real rural per capita GDP) surpasses 8514.27 RMB, the development of inclusive finance shows an increasing marginal effect on both poverty alleviation and household income promotion[14]. In summary, as economic environments and policy support change over time, compounded by variations in household income levels, the impact of household borrowing on family income is uncertain. Consequently, it is necessary to consider the current income characteristics of Chinese rural households and the latest rural revitalization support policies in China, updating research conclusions with the most recent data.

(3) Internet usage facilitates rural household production by influencing credit access. The use of the internet has a significant positive impact on the level of credit access for rural households, enhancing both the availability and scale of credit. Across different loan channels, internet usage significantly increases the level of formal and informal credit access for rural households[16]. Internet usage promotes green production among rural households by enhancing credit acquisition[17]. However, internet usage is only one dimension of digital literacy; other dimensions, such as digital communication and content creation, may also enhance credit acquisition capabilities and, consequently, impact income. The existing literature lacks research on these aspects. Therefore, this paper employs the latest data to comprehensively examine the level of digital literacy among rural households and analyzes the influence of digital literacy on credit access and income.

The marginal contribution of this paper may be as follows: First, based on UNESCO’s “Global Digital Literacy Framework,” and in conjunction with the economic development reality of rural China, this paper examines the influence of digital literacy on rural household income across seven dimensions: device and software operations, information and data literacy, communication and collaboration, digital content creation, safety, problem-solving, and career-related competences. Second, while existing literature has theoretically analyzed the pathways of “internet use affecting credit access” and “credit access influencing rural household production,” there is a scarcity of research discussing the pathway of “digital literacy affecting household income through credit access.” Utilizing the latest 2023 survey data from rural China, this paper elucidates the intrinsic mechanism by which digital literacy influences household income via credit access, providing a roadmap for income growth in the digital economy era. Third, while verifying that the enhancement of digital literacy contributes to rural household income growth, this paper also analyzes the heterogeneity of the income-increasing effect of digital literacy—specifically, this effect varies with differences in income levels, educational attainment, and gender.

The structure of the paper is as follows: Section 1 provides the research background, a review of the existing literature, and the significance of this study. Section 2 outlines the theoretical analysis and research hypotheses. Section 3 describes the data sources, variable definitions, and model specification. The structure of the paper is as follows: Section 1 provides the research background, a review of the existing literature, and the significance of this study. Section 2 outlines the theoretical analysis and research hypotheses. Section 3 describes the data sources, variable definitions, and model specification. Section 4 reports the empirical results and analysis, encompassing baseline regression outcomes and discussion, endogeneity tests, and robustness checks. Section 5 is dedicated to the mechanism analysis. Section 6 conducts heterogeneity analysis. Section 7 concludes and discusses policy implications.

# 2. Theoretical Analysis and Research Hypotheses

**2.1 The Impact of Digital Literacy on Household Income**

Drawing on Schultz’s human capital theory, higher labor quality correlates with greater labor efficiency, suggesting a positive relationship between human capital investment and income returns. In the digital era, workers’ digital literacy has emerged as a crucial form of new human capital, which, when enhanced among rural households, can increase their production efficiency[18], thereby raising income levels. Digital literacy encompasses areas such as device and software operations, information and data literacy, communication and collaboration, digital content creation, safety, problem-solving, and career-related competences. Specifically, stronger digital device operation skills enable households to overcome barriers to digital technology access, using digital tools more effectively and fully. Higher digital resource acquisition literacy allows for better utilization of market information and production factors, reducing job search and business operation costs. Enhanced digital communication and content creation skills empower households to proactively harness digital technologies for profit, such as engaging in information exchange, product promotion, social capital expansion, and human capital enhancement through online platforms.Greater digital safety literacy fosters risk awareness and capitalizes on opportunities in new digital technologies and platforms. Stronger problem-solving skills with digital tools improve work efficiency.

In summary, the improvement of digital literacy aids households in better applying advanced agricultural technologies, timely accessing market information, and effectively utilizing e-commerce platforms, thereby increasing agricultural income through enhanced production efficiency, reduced operation costs, and expanded product market reach. Additionally, it helps households to improve digital skills, expand social networks, enhance risk awareness, and increase job search and career competitiveness,which in turn boosts non-agricultural employment and entrepreneurial opportunities, reduces related costs, and diversifies household income sources.

Based on the above analysis, the following hypothesis is proposed:

Hypothesis 1: Higher digital literacy significantly increases rural household agricultural income, non-agricultural income, and total income.

## 2.2 Digital literacy influences household income by enhancing loan capacity.

Income disparities among farmers are rooted in differences in production costs and initial endowment inputs. Credit funds, as a source of variable inputs, can optimize the initial endowment investment in agricultural production, thereby enhancing agricultural efficiency and reducing income disparities among farmers[19]. In emerging developing countries with a prominent dual economic structure, the contradiction of optimizing the allocation of credit resources between urban and rural areas and across different industries may persist over a certain period.

Traditional formal financial institutions struggle to penetrate rural areas and, to avoid adverse selection and moral hazard, typically impose stringent qualifications on borrowers, leading to the exclusion of farmers. The enhancement of digital literacy allows farmers to utilize digital financial tools, overcoming geographical constraints and accessing collateral-free, rapid-disbursing credit services such as Ant Check Later and Weilidai [20]. In terms of informal lending, increased digital literacy breaks through the geographical limitations of farmers’ social networks, expanding their circles of friends and enhancing the likelihood of private lending [21]. In formal lending, with improved digital literacy, farmers leave more digital financial usage traces, enabling banks to assess individual creditworthiness based on digital financial information and to observe informal lending behaviors to mitigate information asymmetry in the lending process. Consequently, digital literacy can enhance farmers’ credit access, alleviate financial constraints, and alter initial endowments. Access to more credit funds can improve agricultural infrastructure investment, reduce barriers to technology adoption due to insufficient funds, and increase investment in agricultural and non-agricultural operations, production, and sales, thereby promoting income growth for farmers. Therefore, the hypothesis is proposed:

**Hypothesis 2:** Digital literacy promotes household farmer income increase through enhanced credit access.

## 2.3 Heterogeneity of Household Characteristics

A notable question arises: does the advantage of digital literacy trigger a positive feedback mechanism, leading to a “winner-takes-all” scenario, or what is commonly referred to as the “Matthew effect”? Theoretically, individual endowment disparities may result in unequal application of digital technology, which could, in turn, engender new forms of inequality within the digital economy[22]. Regarding individual endowments, classical growth theory posits that physical and human capital are core determinants of growth. Consequently, the impact of digital literacy on the income growth of farmers is contingent, to some extent, on their own endowments of physical and human capital.

Compared to low-income farmers, high-income farmers possess greater physical capital, which can be invested in reproductive activities and the expansion of production scale. This enables high-income farmers to derive greater economic benefits from the application of digital technology.

The role of human capital in this context remains ambiguous. On one hand, human capital influences the capability and cognition of digital technology application; individuals with higher levels of education may better master the use of digital technologies [23]. On the other hand, individuals with varying educational backgrounds may prefer different digital application scenarios. The network effects and economies of scale in the digital economy have created more profitable models for those with lower educational attainment. For instance, content creators in areas such as short videos and live streaming have achieved various monetization paths by attracting a large fan base and traffic, including advertising and product endorsements. The Long Tail Effect can aptly explain this phenomenon, describing how a large number of low-unit-value products or services, which individually may not be bestsellers, can collectively form a significant market share, with the aggregate value of these ‘long tail’ items potentially rivaling or exceeding that of the ‘head’ (bestsellers). Farmers’ participation in markets that align with their cognitive profiles can yield higher returns. The low barriers to entry and high inclusivity of the digital economy have, to some extent, mitigated the traditional impact of educational background on income.

In rural China, traditional norms persist, characterized by the inheritance of physical capital by males and the undervaluation of female human capital accumulation. Such structural gender disparities may induce heterogeneity in the impact of digital literacy on farmers' income. Within the framework of traditional gender division of labor, male farmers tend to engage in large-scale agricultural production—including grain cultivation, cash crop farming, and cross-regional economic activities—whereas female farmers are primarily concentrated in small-scale household agriculture and local service sectors [24].However, internet usage can mitigate gender discrimination by influencing traditional perceptions and enhancing women’s skills and experience[25].The digital economy boosts women’s autonomy and mitigates the “marriage” and “motherhood” penalties[26]. Digital literacy enhances rural women’s workforce engagement, increasing their human and social capital to reduce the income gap with rural men.

Hypothesis 3: The marginal impact of digital literacy on household income varies according to different levels of farmers' income, education, and gender.

# 3. Materials and Methods

## 3.1. Model Setting(

To estimate the impact of farmers’ digital literacy on their income, this study employs the Ordinary Least Squares (OLS) method as the benchmark regression. Considering the issue of heteroscedasticity in cross-sectional data that may lead to biased parameter estimates, robust standard errors are incorporated in the regression analysis.The model is configured as follows:

${lnincome}_{i}=\alpha_{0}+\alpha_{1}{DL}_{i}+\alpha_{2}X_{i}+\varepsilon_{i}$	(1)

where *i* represents various farm households; *income_i_* denotes the total income of farm household *i*.Here, the log form of total income is used in the estimation; *DL* denotes the digital literacy of farm household *i*, which is the explanatory variable of interest in this paper.Based on this regression model, the parameter $\alpha_{1}$ can be utilized to identify the impact of the digital literacy level of rural households on their income.$X_{i}$represents the set of control variables, encompassing personal, household, and regional attributes;$\alpha_{0}$is the constant term;$\varepsilon_{i}$is the error term.

## 3.2. Data Sources and Variable Selection

### 3.2.1 Source of Data

The data were collected from January to July 2023, initiated by the Rural Revitalization Research Institute of Beijing Technology and Business University in collaboration with 14 institutions, including the Department of Agriculture and Rural Affairs of the Ministry of Finance, the Institute of Fiscal Science of the Ministry of Finance, Anhui University of Finance and Economics, Anhui Normal University, Qingdao University, Jinan University, Jiangxi Normal University, and Northwest Normal University.

Survey Target: The survey was conducted among village party secretaries or village heads , and ordinary villagers in rural administrative villages.

Survey Content: The focus was on digital rural development and shared prosperity in rural areas.

Survey Methodology: The Department of Agriculture and Rural Affairs of the Ministry of Finance selected 30 provinces, with 30 administrative villages randomly chosen from each province. The provincial departments of finance were responsible for distributing and collecting the questionnaires. Universities and research institutions combined the survey with college students' social practice credits and provided economic and academic incentives to encourage student participation. Surveyors were then selected based on the required survey regions, with clear specifications on the target respondents and the number of surveys to be conducted.

Quality Assurance of the Survey: The questionnaires distributed by the Ministry of Finance were primarily completed through administrative means, with the content mainly derived from statistical data reported by village committees. To ensure data quality, surveyors were required to record audio, take photos with respondents, and provide location information of the survey sites. The project team conducted random checks on 50% of the questionnaires. Only after these procedures were the questionnaires considered valid.

A total of 1,400 questionnaires were distributed, with 1,283 effectively returned, yielding an effective response rate of 91.64%. The screening criteria included: (1) eliminating extreme outliers; (2) discarding samples with missing basic information; and (3) excluding samples that failed the quality review.

### 3.2.2 Dependent variable

The dependent variable is the total income of farmer’s household, encompassing operational, wage, property, and transfer incomes. For empirical analysis, the variable is logarithmically transformed to enhance precision.

### 3.2.3 Core explanatory variable

The core explanatory variable is the digital literacy. The document "A Global Framework of Reference on Digital Literacy Skills for Indicator" ^[5]^by United Nations Educational,Scientific and Cultural Organization(UNESCO) points out that the digital literacy framework includes seven areas of literacy:Devices and software operations,Information and data literacy,Communication and collaboration,Digital content creation,Safety,Problem solving,Career-related competences.

Drawing on the theoretical framework of the “Global Digital Literacy Framework,” this paper constructs a secondary indicator system comprising 12 indicators, tailored to the economic characteristics of rural China, as detailed in Table 1. The empirical analysis employs an equal-weighting scheme for the baseline regression to aggregate the digital literacy levels of farm households. Subsequently, an entropy-weighted approach is utilized to assign weights and determine the digital literacy levels, thereby assessing the robustness of the equal-weighting results.

### 3.2.4 Other control variables

Control variables include Householder's Characteristic Variables, Household's Characteristic Variables, and Regional Characteristic Variables. The definitions, measurement methods, and descriptive statistics of each variable are provided in Table 2.

The control variables encompass three categories: characteristics of the household head, family characteristics, and regional characteristics. Specifically, the household head characteristics include the gender, age, marital status, and education level of the household head; the family characteristics of rural households include the household size,labor force size,operate business,land area,household savings.The regional characteristics include political accessibility, transport accessibility, and village per capita income.In the empirical model, the Village Per Capita Income is logged.

**Table 1. Digital Literacy Skills for Indicator.**

| **Competency areas** | **Definition** |
| --- | --- |
| Devices and software operations | Possessing a smartphone = 1, otherwise = 0. |
|  | Home broadband installation: Installed = 1, Not installed = 0. |
|  | Household broadband satisfaction is measured on a scale from 1 to 5, with 1 indicating very dissatisfied and 5 indicating very satisfied. |
| Information and data literacy | Internet shopping participation yes= 1, no=0. |
|  | Digital payment capability: Capable = 1, Incapable = 0. |
| Communication and collaboration | Internet communication and collaboration capability: Capable = 1, Incapable = 0. |
| Digital content creation | Adoption of online marketing tactics: Adopted = 1, Not adopted = 0. |
|  | Engagement in live-streaming sales: Engaged = 1, Not engaged = 0. |
| Safety | The Cybersecurity Awareness Scale ranges from 1 to 5, with a score of 1 indicating a lack of familiarity with online anti-fraud knowledge, and a score of 5 representing a comprehensive understanding of online anti-fraud knowledge.. |
| Problem solving | Resolution of production issues via internet: Capable = 1, Incapable = 0. |
| Career-related competences | Engagement in online agricultural product sales: Yes = 1, No = 0. |
|  | Extent of internet technology application in agriculture and livestock: None = 0, Low = 1, High = 2. |

**Table 2. Definition of key variables and descriptive statistics.**

| **Classification** | **Variable Name** | **Definition** | **Mean** | **SD** |
| --- | --- | --- | --- | --- |
| Dependent variables | Total net income | Net annual household income | 114419 | 155765 |
| Core explanatory variables | Digital literacy | Based on the Digital Literacy Skills for Indicator , calculate the weighted score. | 1.011 | 0.207 |
| Control variables | Gender | Householder's gender: Male = 1, Female = 0 | 0.697 | 0.460 |
| Householder's Characteristic Variables | Age | Age of the Householder | 43.84 | 10.99 |
|  | Marital status | Married = 1; Other = 0 | 0.881 | 0.324 |
|  | Educational level | No primary school =1, primary school = 2, junior high school=3, high school or technical secondary school =4, vocational school=5, junior college =6, undergraduate =7, postgraduate =8. | 4.766 | 1.584 |
| Household's Characteristic Variables | Household size | Number of people living together in the household | 4.039 | 1.671 |
|  | Labor Force Size | Number of Laborers in Households | 2.423 | 1.142 |
|  | Operate business | Yes=1,No=0 | 0.355 | 0.479 |
|  | Land area | households' land area | 12.95 | 92.85 |
|  | Household Savings | Yes=1,No=0 | 0.663 | 0.473 |
| Regional Characteristic Variables | Political Accessibility | Distance from Residence to County Government (kilometers) | 23.03 | 21.15 |
|  | Transport Accessibility | Distance from Residence to the Nearest Railway Station (kilometers) | 43.76 | 45.08 |
|  | Village Per Capita Income | Village per capita annual disposable income (in yuan) | 18675.215 | 15509.783 |

### 3.2.5 Mechanism variable.

In this study, the mechanism variable is loan capacity, operationalized as a binary variable based on survey responses to the question “Does the household have a loan?” A “yes” response is assigned a value of 1, indicating loan capacity, whereas a “no” response is assigned a value of 0, indicating the absence of loan capacity. Furthermore, the count of credit access channels is employed to gauge loan capacity, thereby assessing the robustness of the empirical mechanism test.

# 4. Estimation Results and Analysis

## 4.1 Benchmark regression results

To examine the impact of digital literacy on household farmer income, this paper employs ordinary least squares (OLS) regression based on Equation (1). Initially, the Variance Inflation Factor (VIF) is calculated, with a maximum value of 1.81, which is well below the threshold of 10, indicating no multicollinearity concerns. Subsequently, robust standard errors are utilized to address potential heteroscedasticity. Regression results are presented in Table 3.

Columns (1)–(2) in Table 3 below analyze the overall impact of digital literacy on income of a farmer’s household using the regression method that gradually adds core explanatory variables, control variables. The adjusted R-squared increases from 0.050 to 0.205, indicating that the addition of control variables improves the model’s goodness of fit. Moreover, the coefficients of digital literacy are all significantly positive, indicating that the improvement of digital literacy can significantly increase the overall income of residents, and hypothesis 1 is true. It can be seen from column (2) that the coefficient of digital literacy is 0.429, which is significantly positive at the 1% significance level, indicating that each unit of increase in digital literacy will increase farmer’s household by 39.5%. This indicates that in China’s all-out development of the digital economy, digital literacy has emerged as a crucial human capital component for enhancing farmer incomes.

**Table 3.The effect of digital literacy on farm household income.**

| **Variables** | **Income** | |
| --- | --- | --- |
|  | OLS(1) | OLS (2) |
| Digital literacy | 0.825^***^ | 0.395*** |
|  | (0.104) | (0.0997) |
| Gender |  | 0.0642 |
|  |  | (0.0420) |
| Age |  | -0.00163 |
|  |  | (0.00238) |
| Marital status |  | 0.208*** |
|  |  | (0.0782) |
| Educational level |  | 0.0779*** |
|  |  | (0.0138) |
| Household size |  | 0.00920 |
|  |  | (0.0183) |
| Labor Force Size |  | 0.0994*** |
|  |  | (0.0248) |
| Operate business |  | 0.175*** |
|  |  | (0.0424) |
| Land area |  | 0.0124** |
|  |  | (0.00619) |
| Household Savings |  | 0.194*** |
|  |  | (0.0381) |
| Political Accessibility |  | 0.00351*** |
|  |  | (0.00111) |
| Transport Accessibility |  | -0.00153*** |
|  |  | (0.000493) |
| Village Per Capita Income |  | 0.200*** |
|  |  | (0.0350) |
| Constant | 10.49^***^ | 7.979*** |
|  | (0.104) | (0.380) |
| Observations | 1242 | 1242 |
| R-squared | 0.051 | 0.213 |
| adj. R-squared | 0.050 | 0.205 |

Robust standard errors in parentheses

*** p<0.01, ** p<0.05, * p<0.1

## 4.2. Endogeneity Test and Robustness Analysis

### 4.2.1. Endogeneity Test

The baseline OLS estimation may be subject to two types of endogeneity biases that could lead to biased results. First, high-income farmers are more likely to purchase smart devices and participate in paid digital training, which may result in reverse causality bias. Second, there may be omitted variable bias.

This study employs the Instrumental Variable (IV) approach to address reverse causality bias. The IV strategy in this study draws on the design of historical exogenous variables proposed by Dafeng Xu (2017)[27], constructing IVs based on the dual exogenous structure of geographical endowments and historical policies. The first instrumental variable (IV1) selected in this study is the distance from farmers' residences to the administrative center. The location of township-level administrative centers is determined by county-level master plans and has remained stable for a long time since the "township merger policy" in the 1990s. The farther the distance to the township administrative center, the lower the convenience for farmers to access offline services, and the higher their dependence on digital infrastructure and online digital resources—this indirectly affects digital literacy. The second instrumental variable (IV2) is whether a village has 5G coverage. In 2019, national-level planning launched 5G planning and construction, which did not take farmers' income as the criterion for 5G coverage, making it an exogenous variable derived from historical policies. Villages with 5G coverage are more conducive to farmers participating in online digital activities to improve their digital literacy.

The results of the Two-Stage Least Squares (2SLS) estimation are presented in Table 4.The first-stage regression results show: (1) The overall F-statistic = 14.95 (P<0.001), which is greater than the empirical critical value of 10, indicating that the two IVs jointly have a significant explanatory power for digital literacy, satisfying the strong correlation assumption; (2) The Cragg-Donald Wald F-statistic is 23.67. For the scenario of "1 endogenous variable and 2 instrumental variables," the Stock-Yogo 10% maximum IV size critical value is 19.93. The current F-statistic exceeds this critical value, avoiding estimation bias caused by weak instruments; (3) The Sargan statistic = 1.23 (P=0.267>0.05), failing to reject the null hypothesis that "all instrumental variables are exogenous." Thus, the exogeneity test of the IVs is passed.

The 2SLS estimation results show that the IVs effectively correct endogeneity bias, and the core conclusions are robust. The coefficient of digital literacy is 1.520, which is significant at the 1% level. Compared with the baseline OLS, the coefficient of digital literacy in 2SLS is significantly larger—this verifies the conjecture that "reverse causality leads to OLS underestimating the true effect," and the IVs correct this bias.

**Table 4.Results of Endogeneity Test.**

| **Variables** | **First-Stage Regression:Digital literacy** | **Second-Stage Regression:Income** |
| --- | --- | --- |
| Digital literacy |  | 1.520*** |
|  |  | (0.543) |
| IV1 | 0.005 |  |
|  | （0.008） |  |
| IV2 | 0.08*** |  |
|  | （0.011） |  |
| Control variables | Yes | Yes |
| Constant | 0.761*** | 7.086*** |
|  | (0.101) | (0.538) |
| Observations | 1242 | 1242 |
| R-squared | 0.157 | 0.130 |

Robust standard errors in parentheses

*** p<0.01, ** p<0.05, * p<0.1

In order to enhance the precision of causal inference, this paper further employs Propensity Score Matching (PSM) to address endogeneity issues.

As can be seen from Table 5, the propensity score matching estimates are close to the benchmark model, further validating the conclusions that digital literacy has a positive impact on farmers' income.

**Table 5.Average Treatment Effect on the Treated (ATT) Estimate.**

| **Treatment Effect** | **Treated** | **Controls** | **Difference** | **S.E.** | **T-stat** |
| --- | --- | --- | --- | --- | --- |
| Pre-matching | 11.416 | 11.161 | 0.254*** | 0.043 | 5.79 |
| nearest-neighbor matching | 11.414 | 11.307 | 0.107 ** | 0.047 | 2.26 |
| radius matching | 11.414 | 11.305 | 0.108** | 0.047 | 2.29 |
| kernel matching | 11.414 | 11.304 | 0.110** | 0.047 | 2.33 |

The aforementioned results indicate that the coefficient signs of the impact of digital literacy on household income, as obtained through the instrumental variable(IV) method and propensity score matching(PSM) , are consistent with those of the benchmark model. This suggests that, after addressing endogeneity concerns, digital literacy continues to exert a significant positive effect on household income.

### 4.2.2. Robustness Test

1.Replacing the dependent variable. This paper employs household total income as the dependent variable in the baseline regression. Considering that total household income may be influenced by family size, this study replaces the dependent variable with household per capita income and re-conducts the empirical test. As shown in the first column of Table 8, digital literacy continues to have a significant positive impact on household per capita income.

2.Weight adjustment. In the baseline regression, an equal-weighting method is used to calculate the digital literacy level of households. The equal-weighting approach is a widely adopted method in the literature for constructing composite indices. This paper further employs the entropy method to recalculate the weights of the digital literacy indicators and conducts another empirical test, with the results presented in the second column of Table 8. The regression findings indicate that digital literacy still exerts a significant positive impact on household income.

These tests demonstrate the robustness of the baseline regression results, confirming that digital literacy has a pronounced positive effect on household income.

3.Substituting the Core Explanatory Variable. In the preceding analysis, a multidimensional composite index was employed to measure digital literacy. To avoid potential biases arising from the method of index synthesis, this study re-examines the empirical relationship using a single indicator—“Internet usage”—as a proxy for digital literacy. The results are presented in column (3) of Table 8. The regression analysis indicates that Internet usage continues to exert a significantly positive impact on household income among rural households.

**Table 6.Robustness test estimation results.**

| **Variables** | **Income** | |
| --- | --- | --- |
|  | (1)Replace the dependent variable | (2)Entropy weight method |
| Digital literacy | 0.334*** | 0.779*** |
|  | (0.104) | (0.160) |
| Control variables | Yes | Yes |
| Constant | 7.823*** | 8.007*** |
|  | (0.384) | (0.373) |
| Observations | 1242 | 1242 |
| R-squared | 0.299 | 0.220 |
| adj. R^2^ | 0.291 | 0.212 |

Robust standard errors in parentheses

*** p<0.01, ** p<0.05, * p<0.1

# 5. Mechanism Test

To test the mechanism by which digital literacy increases household farmer income by enhancing credit access capacity, this paper introduces the Loan Capacity variable. First, digital literacy is regressed on Loan Capacity, with Column (1) of Table 9 showing a significant positive impact of digital literacy on Loan Capacity. Subsequently, both digital literacy and Loan Capacity are regressed on farmer income, with Column (2) of Table 9 indicating that Loan Capacity acts as a mediating factor, suggesting that digital literacy increases farmer income by improving Loan Capacity. These results are based on Loan Capacity measured by the “presence of household loans.” Additionally, credit access capacity is also measured by the “number of credit access channels,” with results consistent with those in Table 9.

**Table7. Mechanism Test——Alleviating farmers’loan constraints.**

| **Variables** | **（1）Loan capacity** | **（2）Income** |
| --- | --- | --- |
| Digital literacy | 0.179*** | 0.366*** |
|  | (0.0672) | (0.0988) |
| Loan capacity |  | 0.159*** |
|  |  | (0.0410) |
| Control variables | Yes | Yes |
| Constant | 0.170 | 7.952*** |
|  | (0.230) | (0.382) |
| Observations | 1242 | 1242 |
| R-squared | 0.125 | 0.223 |

Robust standard errors in parentheses

*** p<0.01, ** p<0.05, * p<0.1

Furthermore, this study validates the mediating role of loan capacity through Sobel test and the Bootstrap method. The results of the Sobel test are presented in Table 10: Path c indicates the regression of digital literacy on household income, path a represents the regression of digital literacy on loan capacity, and paths b and c’ denote the joint regression outcomes of digital literacy and loan capacity on household income. The z-value is significant at the 1% significance level.

**Table 8.Mechanism Test: Sobel Test Results.**

| **Variables** | **path c** | **path a** | **paths b and c'** |
| --- | --- | --- | --- |
| Digital literacy | 0.394*** | 0.179*** | 0.366*** |
|  | (0.098) | (0.068) | (0.098 ) |
| Loan capacity |  |  | 0.159 *** |
|  |  |  | (0.041 ) |
| Control variables | Yes | Yes | Yes |
| Constant | 7.978 *** | 0.170 | 9.738*** |
|  | (0.333) | (0.230) | ( 0.181 ) |
| Observations | 1,242 | 1,242 | 1,242 |
| R-squared | 0.213 | 0.124 | 0.222 |
| Adj R-squared | 0.205 | 0.115 | 0.214 |

Robust standard errors in parentheses

*** p<0.01, ** p<0.05, * p<0.1

Utilizing the Bootstrap method with 1000 iterations and a 95% confidence interval, the results in Table 11 indicate that the confidence interval (LLCI = 0.001, ULCI = 0.055) does not include zero, signifying a significant indirect mediating effect with a magnitude of 0.028.

**Table 9.Mechanism Test: Bootstrap Test Results.**

| **Indirect effect of Loan capacity on Income** | | | | | |
| --- | --- | --- | --- | --- | --- |
| Effect | SE | Z | P | LLCI | ULCI |
| 0.028 | 0.013 | 2.06 | 0.040 | 0.001 | 0.055 |
| **Direct effect of Loan capacity on Income** | | | | | |
| Effect | SE | Z | P | LLCI | ULCI |
| 0.366 | 0.101 | 3.60 | 0.000 | 0.167 | 0.565 |

The findings suggest that digital literacy enhances household farmer income by improving credit access capacity, thus validating Hypothesis 2. The cultivation of digital literacy enables farmers to overcome credit constraints imposed by information and technology barriers, expanding credit access channels and increasing credit availability. By leveraging the digital financial data of rural households, credit providers can effectively enhance the efficiency of credit approval, achieve intelligent risk control, and reduce customer acquisition costs. The essence of credit supply and access lies in creditworthiness. On digital platforms, the behavioral data of rural households can be closely integrated with financial services, thereby facilitating the circulation of credit through the information flow of these households.

Specifically, farmers can access material and social capital through internet platforms, which enables them to seek employment or start businesses online. They can generate income by selling their own products, promoting goods on behalf of others, or live-streaming rural life. The data generated from these digital activities serve as the foundation for digital credit assessment on internet finance platforms, thereby further optimizing the credit ratings of rural households. Moreover, internet platforms provide efficient channels for the productive use of credit funds, enhancing the income-generating capacity of rural households. Against the backdrop of China's digital financial development, leading e-commerce platforms have established specialized sections to support agriculture, thereby expanding online sales channels for agricultural products. Meanwhile, social media platforms actively participate in agricultural support initiatives through live-streaming sales and short video promotions. These innovative measures significantly enhance the efficiency and profitability of credit fund utilization among rural households, effectively reducing the risk of default on credit funds and contributing to the optimization of rural households' credit ratings. This, in turn, promotes a virtuous cycle in the supply and access of credit for rural households and provides robust support for the sustainable development of financial services for rural households.

# 6. Heterogeneity analysis

## 6.1 Income level differences

Differences in economic capacity are a key determinant of digital inequality . The development of digital infrastructure has made it possible for low-income groups to access modern digital technologies. Concurrently, as the digital literacy of low-income populations improves, digital usage is increasing. However, in terms of the economic output of digital usage, high-income groups are more likely to engage in productive activities with digital technologies (such as work and study), while low-income groups tend to use digital technologies for leisure activities (such as socializing and entertainment), leading to the emergence of digital inequality. Drawing on this analysis, this paper uses household income as a proxy variable to capture variations in the economic capacity of rural households .

**Table 10.Heterogeneity test of Income level and Income source.**

| **Variables** | **Income** | | | | |
| --- | --- | --- | --- | --- | --- |
|  | **10th percentile** | **25th percentile** | **50th percentile** | **75th percentile** | **90th percentile** |
| Total net income | 0.381*** | 0.305*** | 0.369*** | 0.312** | 0.494*** |
|  | (0.140) | (0.105) | (0.0917) | (0.125) | (0.145) |
| Agricultural income | 1.394*** | 1.271*** | 0.656** | 0.571** | 0.502 |
|  | (0.334) | (0.322) | (0.255) | (0.260) | (0.399) |
| non-agricultural income | 0.130 | 0.104 | 0.300** | 0.259** | 0.214 |
|  | (0.205) | (0.146) | (0.123) | (0.108) | (0.141) |
| Control variables | Yes | Yes | Yes | Yes | Yes |

Specifically, considering the potential heterogeneity in the impact of digital literacy on household income across different income groups, this paper employs quantile regression analysis, selecting five representative quantiles: 10th, 25th, 50th, 75th, and 90th. Table 12 reveals significant differences in the coefficients of digital literacy across quantiles, indicating that the effect of digital literacy on household income exhibits group heterogeneity. Although the impact of digital literacy on total household income is significant and positive across all quantiles, a closer inspection reveals that the coefficient at the 90th quantile is notably higher than at other quantiles. This suggests that while improvements in digital literacy significantly boost income growth for middle- to low-income households, the income growth for the highest-income households is disproportionately favored by digital literacy enhancements.

## 6.2 Income source differences

This indicates that enhancing digital literacy is beneficial for increasing the agricultural income of low-income households, which could help narrow the income gap within rural households. For non-agricultural income, digital literacy’s marginal contribution is greatest for middle- to high-income groups, with coefficients at the 50th and 75th quantiles significantly higher than at other quantiles. These findings suggest that digital literacy can significantly increase agricultural income for low-income groups and non-agricultural income for middle- to high-income groups. This provides insights for both households and policymakers on digital literacy cultivation: focusing on agricultural-related digital skills for low-income groups and non-agricultural industry-related digital skills for middle- to high-income groups, as this targeted skill development offers the greatest marginal contribution to household income enhancement.

## 6.3 Educational level differences

Several studies have demonstrated that individuals with higher levels of education not only possess stronger digital usage skills but also exhibit higher participation rates in digital economic sectors such as e-commerce . However, the low barriers to entry and high inclusivity of the digital economy may afford the less educated a “catch-up advantage,” enabling them to enter digital industries that align with their cognitive profiles. Particularly in China, e-commerce platforms targeting the “lower-tier market” have generated numerous employment opportunities for individuals with lower human capital. This paper categorizes households into low human capital groups (below high school level) and high human capital groups (high school level and above) based on the educational attainment of the household head to examine the heterogeneous characteristics of education.

The regression results in Table 13 indicate that digital literacy promotes income growth among both low-human-capital and high-human-capital rural households, with a more pronounced effect on the latter.

This intriguing phenomenon is unfolding in rural China, where individuals of varying educational backgrounds can conveniently engage in live-streaming sales and attract a specific fan base. Influencers with lower educational attainment, often referred to as “grassroots celebrities,” can achieve sales volumes comparable to those of “elites” or “stars.” In China, the expansion of digital channels has provided broader space for public discourse across different social strata, while the rapid development of the digital economy has created diversified new pathways to income generation for a wide range of groups.

**Table 11.Heterogeneity test of educational level.**

| **Variables** | **Income** | |
| --- | --- | --- |
|  | **Low human capital** | **High human capital** |
| Digital literacy | 0.344*** | 0.376** |
|  | (0.117) | (0.177) |
| Control variables | Yes | Yes |
| Constant | 8.043*** | 6.566*** |
|  | (0.506) | (0.683) |
| Observations | 717 | 525 |
| R-squared | 0.251 | 0.191 |
| Adj R-squared | 0.237 | 0.171 |

Robust standard errors in parentheses

*** p<0.01, ** p<0.05, * p<0.1

## 6.4 Gender differences

Ordinary Least Squares (OLS) regressions are conducted separately for male and female samples, with results presented in Table 14. Digital literacy exerts a significantly positive impact on the income of both men and women. However, its income-enhancing effect is more pronounced among rural men than among rural women. Despite the fact that digital literacy provides rural women with greater access to employment opportunities, traditional gender role divisions and family responsibilities constrain their participation in high-income sectors. Compared to men, women's entrepreneurship and employment are more concentrated in low-income fields, such as handicraft production and family-based side businesses, which have relatively limited income growth potential.

**Table 12.Heterogeneity test of gender.**

| **Variables** | **Income** | |
| --- | --- | --- |
|  | **Male** | **Female** |
| Digital literacy | 0.405*** | 0.374* |
|  | (0.119) | (0.190) |
| Control variables | Yes | Yes |
| Constant | 8.239*** | 7.469*** |
|  | (0.476) | (0.638) |
| Observations | 866 | 376 |
| R-squared | 0.227 | 0.230 |
| Adj R-squared | 0.216 | 0.204 |

Robust standard errors in parentheses

*** p<0.01, ** p<0.05, * p<0.1

## 6.5 Differences in digital literacy dimensions

To delve into which aspects of digital literacy have the greatest impact on income, this paper regresses the seven dimensions of digital literacy on household farmer income. Table 15 reveals the impact of each domain of digital literacy. The coefficients for Devices and Software Operations, Information and Data Literacy, and Career-related Competences are significantly positive. This indicates that the primary domains of digital literacy driving income growth for farmers are basic operations of digital devices, information search, and the application of internet technologies related to agriculture. Farmers exhibit significant deficiencies in digital collaboration, digital security, and integrating digital content to solve problems.

**Table 13.Heterogeneity test of digital literacy dimension.**

| **Variables** | **Income** |
| --- | --- |
| Devices and software operations | 0.092^**^ |
|  | (0.046) |
| Information and data literacy | 0.214^***^ |
|  | (0.072) |
| Communication and collaboration | 0.023 |
|  | (0.072) |
| Digital content creation | -0.153 |
|  | (0.118) |
| Safety | -0.017 |
|  | (0.024) |
| Problem solving | -0.105^**^ |
|  | (0.048) |
| Career-related competences | 0.273^***^ |
|  | (0.054) |
| Control variables | Yes |
| Constant | 7.955^***^ |
|  | (0.392) |
| Observations | 1242 |
| R-squared | 0.234 |
| adj. R-squared | 0.222 |

# 7. Conclusions and policy implications

With the growth of the digital economy, rural residents can enhance their income levels by improving their digital literacy. Utilizing data from a 2023 survey of Chinese rural households, this paper employs quantile regression, instrumental variable methods, and propensity score matching (PSM) to examine the impact of digital literacy on household income. The findings are as follows: (1) Digital literacy significantly increases overall household income levels, a conclusion that is robust. (2) Digital literacy enhances farmers’income by improving their capacity to access credit. (3) Digital literacy significantly boosts agricultural income for low-income groups and non-agricultural income for middle- to high-income groups, with the greatest marginal impact on the highest-income households. (4) Digital literacy promotes income growth across individuals with varying levels of educational attainment, yet its impact is weaker among those with lower levels of education. Additionally, digital literacy enhances income growth for both genders, but its positive effect on male income is greater than that on female income.(5) The current dimensions of digital literacy that primarily drive income growth for farmers are basic digital device operations, information search, and the application of internet technologies related to agriculture.

Based on the research, the following policies are proposed: (1) Digital skill development for farmers: Tiered and categorized training will be jointly conducted by county-level governments in collaboration with local agricultural universities and agricultural-related enterprises, covering the following three aspects: First, training on digital collaboration capabilities. Village-level cooperatives will take charge of online order management; leading enterprises will be responsible for online sales and transportation management; and small-scale farmers will handle offline production. Agricultural universities and enterprises will formulate the full-process collaboration plan and provide corresponding training. Second, training on digital security capabilities. Village-level cooperatives and leading enterprises will deliver case-based teaching to farmers, focusing on identifying agricultural-related telecom fraud and protecting the privacy of agricultural production data. Third, training on digital content integration capabilities. The government will cooperate with leading e-commerce enterprises to develop APPs or websites, which will be used to analyze and release market demand. A unified online platform will integrate all entities in the agricultural product supply chain and sales chain, providing farmers with training and services covering all links of e-commerce.

Meanwhile, a 6-month post-training follow-up mechanism will be established, aiming to increase the usage rate of digital tools among low-income groups by more than 30%.

(2) Connection between digital credit and digital industries: First, expand access to digital credit. The government will promote financial institutions such as rural credit cooperatives and MYbank to launch pro-farmer digital credit, which is a credit loan based on farmers’ production data and the pledge of pre-sold agricultural product orders. Second, guide the targeted investment of credit funds. The government and financial institutions will give priority to supporting the settlement of agricultural product e-commerce industrial parks, the IoT transformation of greenhouse farms, and the rural live-streaming economy. They will also invest funds to support the incubation of farmers’ live-streaming accounts for product promotion.

(3) Improvement of digital literacy among vulnerable groups: First, the government will guide public perception: Through village radio broadcasts, short videos, and door-to-door lectures, it will popularize cases of "increasing income via digital tools", such as stories of local rural women increasing their income by selling handcrafted products through e-commerce. Second, tiered training: For groups with education level of primary school or below, an illustrated Rural Digital Skills Manual (with a function of scanning QR codes to watch practical operation videos) will be developed, and "digital assistance positions" will be set up in village service centers (staffed with college-graduate village officials or volunteers to provide one-on-one guidance). The government and enterprises will launch "Women’s Digital Skills Classes" for rural women, focusing on e-commerce operation of handcrafted products and live-streaming of parent-child-oriented agricultural products.

This study has achieved certain results in terms of theoretical analysis and empirical research, but there is still room for improvement. Future research plans to adopt appropriate panel data to conduct an in-depth exploration of the temporal impact of digital literacy improvement on residents' income, so as to enhance the universality of the conclusions.

# References.

1. Zhou, Y., Zhou, W. (2024). The impact of digital inclusive finance on the supply of trade credit to rural households. Nankai Economic Studies, (02), 83-99.
2. Palvia, P., Baqir, N., Nemati, H., 2018. ICT for socio-economic development: a citizens’perspective. Inf. Manag. 55 (2), 160–176.
3. Chen, Z., Cui, R., Tang, C., & Wang, Z. (2024). Can digital literacy improve individuals' incomes and narrow the income gap?. Technological Forecasting and Social Change, 203, 123332.
4. Evans, O., 2018. Digital agriculture: mobile phones, internet agricultural development in Africa. Actual Problems of Economics 7-8 (205–206), 76–90.
5. Correa, T., Pavez, I., Contreras, J., 2020. Digital inclusion through mobile phones?: a comparison between mobile-only and computer users in internet access, skills and use. Inf. Commun. Soc. 23 (7), 1074–1091.
6. Liu, S., Wei, B. H., & Su, K. Y. (2020). Can internet use improve rural households' access to credit? An empirical study based on CFPS panel data. Journal of Economic Theory and Economic Management, (07), 58-72.
7. Czernich, N., Falck, O., Kretschmer, T., & Woessmann, L. (2011). Broadband infrastructure and economic growth. The Economic Journal, 121(550), 505–532.
8. Luo, Q. F., Zhao, Q. F., & Qiu, H. L. (2023). Digital skills and rural household property income—Based on data from the Comprehensive Survey of Rural Revitalization in China (CRRS). Contemporary Economic Management, (07), 54-62.
9. Hartarska, V., Nadolnyak, D., & Shen, X. (2015). Agricultural credit and economic growth in rural areas. Agricultural Finance Review, 75(3), 302-312.
10. Gu, N., & Wang, H. W. (2021). Does rural household borrowing facilitate family income growth? Micro-evidence from rural China. Agricultural Economics and Management, (06), 73-82.
11. Jia, X., Heidhues, F., & Zeller, M. (2010). Credit rationing of rural households in China. Agricultural Finance Review, 70(1), 37-54.
12. Luan, D. X., & Bauer, S. (2016). Does credit access affect household income homogeneously across different groups of credit recipients? Evidence from rural Vietnam. Journal of Rural Studies, 47, 186-203.
13. Hu, Z. Y., & Luo, L. D. (2016). The utility of microcredit in alleviating rural poverty: An analysis based on panel models. Finance Theory and Practice, (03), 10-15.
14. Du, J., Zhang, W. K., & Fan, J. L. (2017). Dual characteristics of rural financial development on farmers' income: An empirical study based on panel threshold model and spatial econometric model. Journal of Huazhong Agricultural University (Social Science Edition), (06), 35-43+149.
15. Gu, N., & Zhang, T. (2019). Inclusive finance development and rural poverty reduction: Threshold, spatial spillover, and channel effects. Journal of Agricultural Technology and Economics, (10), 74-91.
16. Liu, S. L., Zhang, X. M., & Yang, Z. S. (2021). The impact of internet use on rural residents’ income. Journal of Quantitative and Technical Economics, (4), 103-119. doi:10.13653/j.cnki.jqte.2021.04.006.
17. Du, F. J., Zhao, X. Y., Zheng, J., & Zhang, M. Y. (2023). Can digital literacy promote green production among rural households? Evidence from CLES data. World Agriculture, (10), 97-109.
18. Li D, Kojima D, Wu L, et al. Impact of rural households' digital ability on their production efficiency in China[J]. Agribusiness, 2024, 40(1): 139-160.
19. M.Rota(2013).Credit and Ggrowth:Reconsidering Italian Industrial Policy During the Golden Age.European Review of Economic History，17(4):431-451.
20. Li, W. R., & Zhou, S. J. (2023). Transformation of agricultural production methods in China under the digital economy: Mechanisms, contradictions, and resolutions. Journal of Xi'an Jiaotong University (Social Science Edition), (01), 65-73.
21. Li, J. H., & Lu, Q. (2022). The impact of digital finance on rural households' adoption of green production technologies. Journal of Natural Resources, (12), 2470-2486.
22. Van Dijk J. Digital divide: Impact of access. The international encyclopedia of media effects[J]. 2017.
23. Vicente M R, López A J. Assessing the regional digital divide across the European Union-27[J]. Telecommunications Policy, 2011, 35(3): 220-237.
24. He, J., Li, Q., & Zhang, S. (2010). Household gender division of labor and feminization of agriculture: An empirical analysis based on 408 sample households in Jiangsu. Journal of Nanjing Agricultural University (Social Sciences Edition), 10(1), 50-56.
25. Wu, L., Liu, J. J., & Wen, H. Y. (2021). Do rural women's entrepreneurship enjoy a "digital dividend"? An empirical analysis based on CGSS 2015 data. World Agriculture, (08), 53-68+119-120.
26. Xue, Q., Wang, H., Wei, J., & Bai, C. (2024). Does the digital economy improve female autonomy? Structural Change and Economic Dynamics, 193-207.
27. Xu, D. (2017). Acculturational homophily in digital literacy acquisition: Evidence from rural China. Economics of Education Review, 59, 29-42.

# Social Science Foundation Project.

1. Research on the education model of improving Digital Literacy of capital residents under the Strategy of Common Prosperity (CHAA22060).

2. Research on the mechanism, effect and realization path of the high-quality construction of Hainan Free Trade Port driving regional talent revitalization(HNSK(YB)24-21).

3. Research on the new international trade talent training model of Hainan Free Trade Port in the era of digital economy（HNJG2025ZC-46）.

1. See more detail:https://www.gov.cn/lianbo/bumen/202402/content_6933541.htm
2. See more detail:https://www.gov.cn/xinwen/2023-02/27/content_5743484.htm
3. See more detail:https://www.gov.cn/zhengce/2019-05/16/content_5392269.htm
4. See more detail:https://so.html5.qq.com/page/real/search_news?docid=70000021_853604b16c633852&faker=1
5. See more detail:

   http://uis.unesco.org/sites/default/files/documents/ip51-global-framework-reference-digital-literacy-skills-2018-en.pdf
