# Supplementary material for: Digital literacy: The catalyst for credit access and income growth in rural households
Source: PLoS One. 2026 Feb 13;21(2):e0341326. doi: 10.1371/journal.pone.0341326 (PMC12904465; doi:10.1371/journal.pone.0341326)
Supplement: Attachment — Submitted filename: Response_to_Reviewers_auresp_2.docx [file pone.0341326.s004.docx]

Dear academic editors and reviewers,

We would like to extend our sincere gratitude to all of you for your respective valuable comments and constructive suggestions on our manuscript. These insights have been instrumental in enhancing the quality and rigor of our work, and we have carefully addressed each comment from reviewer and academic editor in the revised version, as detailed below.

**Reviewer’s Comments:**

**1.The limitations of using 2023 data are not clearly stated. It is advisable to conduct discussions on whether there were special digital policies in 2023 and the short-term impact of post-pandemic economic recovery on rural digital usage, etc.**

We have provided a detailed explanation of this issue in Section 7 and cited relevant references to demonstrate that the use of 2023 cross-sectional data is unlikely to affect the long-term conclusions. The specific revisions are as follows:

Using only 2023 cross-sectional data may only reflect the short-term impact of both specific digital policies and the post-pandemic economic recovery on rural digital usage. Since the onset of the COVID-19 pandemic, e-commerce has emerged as a primary driver for epidemic control, the resumption of business operations, and the revitalization of the national agricultural and rural economy [40]. Since 2021, the Chinese government has implemented the “Digital Village Development” policy[3] , that was reaffirmed in the “No. 1 Central Document” of 2023 and the “15th Five-Year Plan” recommendations for 2025. This policy exhibits sustained consistency and is set to continue its promotion in the future. Furthermore, the pandemic’s impact on residents’ online consumption habits and digital technology adoption exhibits long-term characteristics[41]. Consequently, while cross-sectional data from 2023 are indeed subject to the dual influences of ongoing policy and post-pandemic economic recovery, these effects are of a long-term nature. This analysis can be validated in future research using time-series data.

2.For the revision, it is supplemented that the survey subjects include ordinary villagers (the original statement only mentioned village cadres). However, it does not address the limitations of data bias, does not discuss the possible administrative statistical bias in the reports of village cadres, and nor does it analyze the potential impact of this bias on the research results.

We have provided a detailed explanation of this issue in Section 7, and the specifics are as follows:

Although our survey respondents are predominantly ordinary villagers, a subset of the data was collected from village cadres. These cadres may have performance incentives that lead to an overstatement of digital inputs and outcomes when reporting on items like digital infrastructure and income. This bias could artificially inflate the estimated effect of digital literacy on farmers’ income. Future research could attempt to correct for this bias by utilizing more objective metrics, such as backend data from telecommunication operators.

3.Many viewpoints lack literature support, and it is recommended to supplement relevant literature. Only 2 articles have been added since the last manuscript, which is unreasonable for such a lengthy discussion in this paper. Upon careful review, many viewpoints are the author's own opinions. For example, the first sentence should be supported by the reference:
Cyberspace Administration of China, et al. (2024). 2024 work priorities for improving national digital literacy and skills. Retrieved from [http://www.gov.cn.](http://www.gov.cn./)
For instance, line 64 can introduce the literature by Liu et al. to elaborate on digital economy and agricultural development: Liu, F., Zheng, D., Zhao, D., & Wen, B. (2026). Spatiotemporal differentiation and evolution of associated networks of China's agricultural innovation ecosystem. Agricultural Systems, 231,104537. [https://doi.org/10.1016/j.agsy.2025.104537.](https://doi.org/10.1016/j.agsy.2025.104537.) Lines 67-68 can draw on Xu’s article for support related to digital finance: Xu, K. (2023). Digital finance, social security expenditures, and rural-urban household income poverty. Evidence based on an area and household level analysis. Finance Research Letters.

We have adopted the reviewer's suggestions, added the references mentioned in the suggestions at the corresponding positions in the original manuscript, and additionally supplemented relevant references and footnotes. Currently, there are 51 references and 9 footnotes, totaling 60.

**Editors notes:**

**We have carefully summarized and responded to the Editor’s suggestions by section.**

**Section 1**

1.1Better add a footnote with a brief introduction to the background.

Thank you for your valuable suggestion. We have adopted the Editor’s suggestions, and please refer to Footnote 1 for the revisions. The specific details are as follows:

The work plan specifies that its core tasks cover 6 areas, focusing on cultivating digital talents, bridging the digital divide, supporting the digital economy, expanding smart life, while strengthening guarantees and cyberspace construction.

1.2Just “the Chinese government” is fine.This is an example that the authors may want to further refine language. Maybe “shall” is better here? I will not identify specific language issues from now on, but please take a close look at the words.

Thank you for your careful review. We have carefully reviewed and revised the entire manuscript to address all language issues.

1.3Please put some of these content to footnote with the link provided in the existing footnote.

We have adopted the Editor's suggestions, and please refer to Footnote 4 for the revisions.

1.4This is a bit messy. Editing?Percent or percentage points? Can we just know the raw numbers?

To make the data more intuitive and clear, we have presented the original data in this revision. The specific revisions are as follows:

As indicated in The Survey and Analysis Report on Digital Literacy in Rural China released by the Chinese Academy of Social Sciences (CASS) in 2021, the overall digital literacy level of Chinese residents remains relatively low. Specifically, rural residents attained a digital literacy score of 35.1, which is 21.2 points lower than that of urban residents. In terms of digital income-generating capacity, rural residents scored 28.8, representing a 27.7-point gap compared with urban residents. Additionally, their score in terms of digital security awareness stood at 28.6, 43.2 points lower than that of their urban counterparts.

1.5Overall, this is an extremely long introductory section. The content itself is fine, but I suggest that the authors should move some of the analysis and literature review to Section 2 (maybe the existing sub-section, maybe a new sub-section). Section 1 should be concise and efficient.

Thank you for your valuable comment. In this revision, we have streamlined the content of Section 1, streamlined and summarized the theoretical analysis and relevant literature, and moved some of the content to Section 2.

**Section 2**

2.1Since each sub-section ends with a hypothesis, which is not a very standard way of organizing Section 2, maybe the authors should consider writing a short introductory paragraph in the beginning of Section 2 to inform readers that there will be one hypothesis at the end of each sub-section. And, briefly, what these hypotheses are about.

In this revision, we have provided the following explanation at the beginning of Section 2:

This section investigates the mechanisms through which digital literacy affects rural household income by testing three hypotheses: (1) whether digital literacy increases agricultural and non-agricultural income; (2) whether it boosts income by enhancing access to credit; and (3) whether its impact varies across households categorized by income level, educational attainment, and gender.

**Section 3**

3.1 I’m not sure why Section 3 begins with a regression model, and then data, and then regresssion model again. How about this: first introduce data (Section 3.2), descriptive stats, and then the model. Also if the authors do anything beyond the simple OLS, this is a good place to discuss these statistical models as well.

In this revision, we have adjusted the order of subsections in Section 3, first introducing “Data Sources and Variable Selection” in Subsection 3.1, followed by “Model Setting” in Subsection 3.2.

3.2Do we have statistics for this table as well? Not only those in Table 2?

We have added specific statistical data consistent with those in Table 2 to Table 1, including mean and standard deviation.

3.3Statistics? The mechanism variable can really be introduced along with other variables in your dataset as well. Just remove this sub-sub-section and, if you think describing it is needed, just add a new paragraph like this.

We have deleted the explanation of the mechanism variable in Section 3, because a detailed explanation of the mechanism variable is provided in Section 5. Thus, it is entirely reasonable to delete this explanation in Section 3.

**Section 4**

4.1No need to test multicollinearity. If you do want to do this, add it in the footnote.

We have deleted the statement regarding the multicollinearity test.

4.2 I won’t use words like “impact” or “effect” if they are not about causal inference. Just say “association” or “correlation”

we have adopted your recommendation and changed it to “association”.

4.3Is it 39.5 percent or 39.5 percentage points? 0.395 seems to be your coefficient, then it should be percentage points, no? Could you check it? (I might be wrong)

Thank you for your question! Let me clarify this point:

This is a semi-log model (the dependent variable is log-transformed, while the independent variable is in raw form). In this specification, the coefficient of the independent variable reflects the approximate percentage change in the dependent variable (not a change in percentage points).

Specifically, the coefficient 0.395 means that a one-unit increase in the independent variable is associated with an approximate 39.5% change in the dependent variable.

Thus, the correct description here is “39.5 percent”.

4.4Just report adj r2.

We have deleted R-squared from all tables and only report adj R².

4.5Thanks for citing my paper – there are definitely other relevant papers as well. But there’s no need to mention names in the text (just numbers), and I think you are referring to the wrong paper. That paper is not about history. If you do want to cite, these papers – https://www.sciencedirect.com/science/article/abs/pii/S0166046218302114 and https://link.springer.com/article/10.1007/s00148-020-00796-0 – are more relevant.

Since your historical IV is also relevant to geography, maybe citing this paper as well: https://www.nber.org/papers/w4483. This is an ancient paper (and is only a working paper), but it is a “gold standard.” Note that this is not very relevant to historical policy IV used in this paper, so don’t overstate its relevance.

We have carefully read the literature mentioned by the Editor, added References 32, 33, and 34 in this revision, and deleted the literature with weak relevance.

4.6Here, it would be very useful to narrate how a policy in the 1990s might not be very relevant to today’s outcome. What’s the context? And, is it possible to present any statistics, even rough ones?

We have added statistical data and references related to the policy context. The specific revisions are as follows:

The first instrumental variable (IV1) selected in this study is the distance from farmers’ residences to the administrative center. The location of township-level administrative centers is determined by county-level master plans and has remained stable for a long time since the “township merger policy” in the 1990s(We have added the link to the official document on the policy context in Footnote 7), the number of townships decreased from 48,250 in 1992 to 35,509 in 2005, with an average annual reduction of approximately 2.2%[35].

4.7Again, the authors need to tell a story about exogeneity – why, in this specific context, the IV is *arguably* valid. None of these tests make the IV valid, and I will actually delete all of these tests except for the first stage F-stat.

We have deleted all of these tests except for the first stage F-stat, and added Footnote 8 and Reference 36 to demonstrate the exogeneity of the instrumental variable here.

4.8I would avoid such terms. I’ll just say things like arguably correct or “we attempt to correct bias using IV” or something like this. Again, IV validity is not testable, and I think the authors should also acknowledge this.

We acknowledge this point and have adopted the Editor’s language expressions.

4.9No need to report constant.Don’t report R-sq for 2SLS (first-stage yes, but not the IV regression)

We have revised in accordance with the suggestion, see Table 4.

4.10I won’t delve into the goal of PSM – just say “we further provide additional evidence of robustness” etc.

We have adopted the Editor’s language expressions.

4.11Write the specific outcome variable the regression uses.Remove constant, r-sq

We have revised in accordance with the suggestion, see Table 6.

**Section 5**

5.1Please identify the name of specific paths, rather than saying them “a b c”.

We have revised in accordance with the suggestion, see Table 8.

Section 6

6.1This is a weird sentence. What’s “this” in the beginning of the section? I normally don’t check language issues, as stated in the beginning of the article, but this one is just too obvious. Please proofread carefully.

We sincerely apologize; a paragraph was accidentally deleted during the revision process, and we have supplemented it in this revision. See Section 6.2, Lines 505 to 509.

6.2Several studies – any literature? I’m not an expert in this field so I have no papers to suggest, but please do a brief lit review if it is claimed that there are several studeis.

We have supplemented References 37 and 38 in this revision.

**Section 7**

7.1I suggest rewriting the concluding section. First, there is no need to present this many policy suggestions. Second, for each suggestion the authors propose, the authors need to link it with the specific empirical results (which table and regression?) supporting this suggestion. Currently these suggestions seem a little bit generic.

We have rewritten the policy recommendations in this revision. First, we summarized and streamlined the policy recommendations; second, we ensured that the policy recommendations correspond to the empirical results. Specifically, Section 7 first lists the empirical conclusions one by one, and then puts forward the recommendations corresponding to these empirical conclusions.

Once again, we would like to thank all of you for your time, expertise, and thoughtful feedback—your insights have been crucial to refining our manuscript. We hope the revised version addresses all your concerns adequately.
